# Structure of the Extracellular Portion of CD46 Provides Insights into Its Interactions with Complement Proteins and Pathogens

**DOI:** 10.1371/journal.ppat.1001122

**Published:** 2010-09-30

**Authors:** B. David Persson, Nikolaus B. Schmitz, César Santiago, Georg Zocher, Mykol Larvie, Ulrike Scheu, José M. Casasnovas, Thilo Stehle

**Affiliations:** 1 University of Tuebingen, Tuebingen, Germany; 2 Centro Nacional de Biotecnología, CSIC, Campus Universidad Autonóma, Madrid, Spain; 3 Laboratory of Developmental Immunology, Massachusetts General Hospital and Harvard Medical School, Boston, Massachusetts, United States of America; 4 Department of Pediatrics, Vanderbilt University School of Medicine, Nashville, Tennessee, United States of America; Harvard Medical School, United States of America

## Abstract

The human membrane cofactor protein (MCP, CD46) is a central component of the innate immune system. CD46 protects autologous cells from complement attack by binding to complement proteins C3b and C4b and serving as a cofactor for their cleavage. Recent data show that CD46 also plays a role in mediating acquired immune responses, and in triggering autophagy. In addition to these physiologic functions, a significant number of pathogens, including select adenoviruses, measles virus, human herpes virus 6 (HHV-6), *Streptococci*, and *Neisseria*, use CD46 as a cell attachment receptor. We have determined the crystal structure of the extracellular region of CD46 in complex with the human adenovirus type 11 fiber knob. Extracellular CD46 comprises four short consensus repeats (SCR1-SCR4) that form an elongated structure resembling a hockey stick, with a long shaft and a short blade. Domains SCR1, SCR2 and SCR3 are arranged in a nearly linear fashion. Unexpectedly, however, the structure reveals a profound bend between domains SCR3 and SCR4, which has implications for the interactions with ligands as well as the orientation of the protein at the cell surface. This bend can be attributed to an insertion of five hydrophobic residues in a SCR3 surface loop. Residues in this loop have been implicated in interactions with complement, indicating that the bend participates in binding to C3b and C4b. The structure provides an accurate framework for mapping all known ligand binding sites onto the surface of CD46, thereby advancing an understanding of how CD46 acts as a receptor for pathogens and physiologic ligands of the immune system.

## Author Summary

The human membrane cofactor protein (MCP, CD46) is expressed on all nucleated cells and serves as a marker that prevents host cells from destruction by the immune system. It functions as a cofactor that helps to inactivate the C3b and C4b molecules, which are central components of the complement system. In addition to its role in regulation complement activation, CD46 is also used by a large number of pathogens, including measles virus and adenovirus, as a receptor to allow these pathogens to attach to the cell surface and initiate an infection. We have determined the three-dimensional structure of the bulk of the extracellular region of CD46 using X-ray crystallography. This structure provides detailed information about the location of previously identified residues that play a role in the interactions with C3b, C4b, and several pathogens, advancing an understanding of the function of the CD46 protein as a host and pathogen receptor. Moreover, the structure also reveals an unexpected, bent conformation of the protein that has implications for how the binding sites are presented at the cell surface.

## Introduction

The human CD46 receptor, also known as membrane cofactor protein (MCP), is present on all nucleated cells [1]. It belongs to a family of proteins known as the regulators of complement activation (RCA), which cluster on chromosome 1q32 [2], [3]. In addition to CD46, the RCA family includes decay-accelerating factor (CD55/DAF), complement receptors 1 (CR1/CD35) and 2 (CR2/CD21), the C4-binding protein, and factor H (FH). CD46 acts as a key regulator in the classical and alternative complement activation cascades of the innate immune system by serving as a cofactor for the factor I - mediated cleavage of C3b and C4b [4]. This process protects host cells from inadvertent lysis by the complement system [3]. The relevance of CD46 has expanded beyond the innate immune system in recent years as it has become clear that CD46 can regulate T-cell immunity, and is in particular able to control inflammation [5]. Consequently, reproductive processes, multiple sclerosis, and inflammatory responses in the brain have all been functionally linked to CD46 [5], [6], [7], [8].

In addition to its role in complement activation and regulation of the adaptive immune response, CD46 is used as a cellular receptor by several viruses and bacteria. Some measles virus (MV) [9], [10] and adenovirus (Adv) [11], [12], [13] strains attach to cells by engaging CD46. In addition, group A *Streptococci* [14], [15], some *Neisseria* strains [16], [17] and human herpes virus 6 (HHV6) [18], [19] all use CD46 as a receptor. While other members of the RCA-cluster are also targeted by viruses [20], [21], the number of pathogens that attach to cells by using CD46 remains unsurpassed. This has led to the description of CD46 as a “pathogen's magnet” [22]. The prominence of CD46 in pathogen interactions may be attributed, at least in part, to the protein's ubiquitous expression in the host. In some cases, interactions with pathogens have also been shown to down-regulate cellular levels of CD46, thereby increasing complement sensitivity of infected cells [23], [24], [25]. A recent study provides evidence for a direct link between CD46 and components of the autophagy machinery [26]. Recognition of pathogens by CD46 is thought to trigger autophagy, which serves as a critical step to control infection. However, some pathogens are known to exploit autophagy in host cells.

Common to all the proteins expressed from the RCA cluster is their modular construction, which is primarily based on concatenated short consensus repeats (SCR) [3]. Each SCR module contains about 60 amino acids that fold into a compact β-barrel domain surrounded by flexible loops [27]. While the modules display high sequence variability, they all contain four conserved cysteine residues that form two disulfide bridges at the top and bottom of the repeat. The number of repeats present in the members of the RCA family ranges from four in CD55 and CD46 to 30 in CD35. Many structures of fragments of RCA family members are known, and they exhibit significant diversity both in their loop structures and also in their interdomain orientation [28], [29]. The four SCRs in CD46 constitute the bulk of its extracellular region. The repeats are connected to a short linker region rich in serines, threonines and prolines (STP region), a single membrane-spanning segment, and a cytoplasmic tail. Alternative splicing generates multiple isoforms of CD46 that all have identical N-terminal repeats but exhibit variation in the STP region and the cytoplasmic tail [30].

The crystal structure of the N-terminal two repeats, SCR1 and SCR2, of CD46 (CD46-2D) revealed essential features of this region, including a pronounced bend between the two repeats and significant flexibility at the interdomain interface [31]. Although CD46-2D is heavily glycosylated, one side of the two-domain fragment was found to be entirely devoid of glycans. Subsequent crystal structures of CD46-2D in complex with the Adv fiber knob [32], [33] and with the MV hemagglutinin [34] demonstrated that both viral attachment proteins bind to this glycan-free surface. In both cases, engagement by the virus leads to “straightening” of the CD46-2D protein into a linear conformation. Furthermore, both viral attachment proteins form contacts with CD46-2D that predominantly involve residues at the SCR1-SCR2 interface. The implications of the structural rearrangement of CD46 upon ligand binding are not understood.

Structural information about the binding of complement proteins C3b and C4b to CD46 is not available. However, biochemical mapping studies strongly implicate domains SCR2, SCR3 and SCR4 in this interaction, with most of the predicted contacts located on SCR3 and SCR4 [35], [36], [37]. Notably, the regions of CD46 that are thought to interact with C3b and C4b overlap but are not identical [37]. As the cellular C3b and C4b proteins as well as HHV6 engage regions that include the SCR3 and/or SCR4 domains, modeling studies have aimed to predict the structure of unknown portions of CD46 in order to provide a basis for the mapping of binding epitopes [37], [38]. Although some features of the SCR domains are conserved and can be predicted with reasonable accuracy, loop regions and interdomain orientations are notoriously difficult to model. These latter features are however central components of the protein and, to a large extent, determine its overall conformation and interaction properties.

In order to advance an understanding of how CD46 interacts with its many ligands, we determined the three dimensional structure of an extracellular segment of CD46 that comprises all four SCR domains (CD46-4D). The structure provides a basis for identifying binding sites for several CD46 ligands that bind to the C-terminal region of the protein. It also reveals an unexpected kink between domains SCR3 and SCR4, which has profound implications for the conformation of CD46 on the cell surface, and for the recognition of its ligands.

## Results

### Structure determination

**Figure 1:**
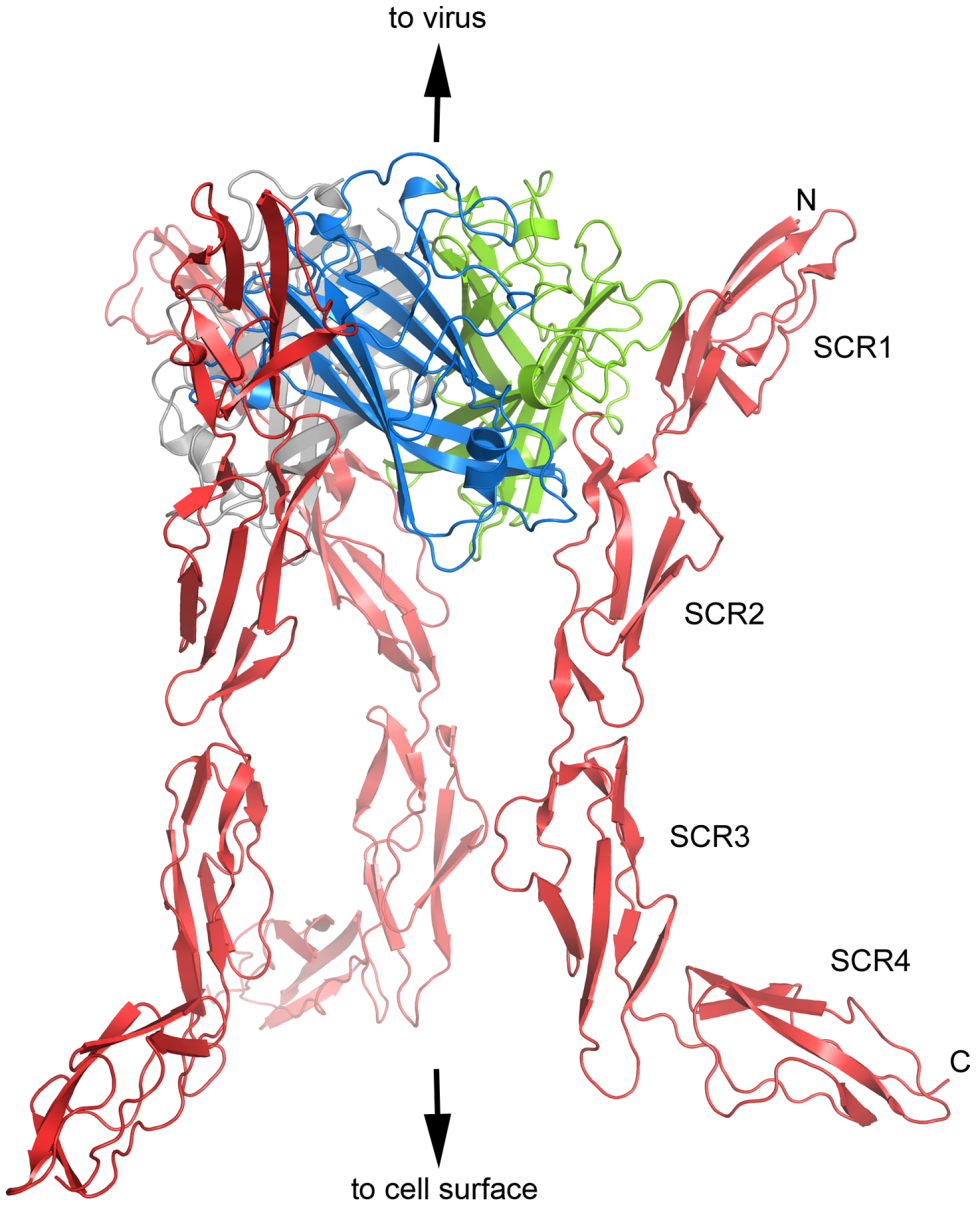
Overall structure of CD46-4D in complex with the Ad11 knob. Ribbon representation of the Ad11 knob trimer, with individual protomers (monomers) shown in blue, green and grey. The knob is bound to three copies of CD46-4D, shown in red. The three-fold axis of the knob lies in a vertical direction. The slightly asymmetric view was chosen to highlight the overall conformation of the CD46-4D molecule on the right hand side.

Glycosylation of CD46 plays an important role in mediating its interactions, at least with some proteins [39]. Proper glycosylation probably also helps to stabilize the overall conformation of the CD46-2D fragment [31]. In order to preserve the glycosylation of CD46-4D, we produced the protein in a mammalian cell line (see Methods). However, efforts to determine the crystal structure of unliganded CD46-4D were unsuccessful, perhaps due to the heavy glycosylation and the known flexibility between domains SCR1 and SCR2 [31]. Although several crystal forms could be obtained, none of these diffracted beyond 15 Å (M. Larvie and T. Stehle, unpublished results). The Ad11 knob, which can easily be crystallized in its unbound form and engages in a high-affinity complex with the SCR1 and SCR2 domains of CD46-2D [32], was then used to form a complex with CD46-4D for crystallization. This strategy produced crystals that diffracted to 2.84 Å resolution, allowing us to trace the polypeptide chains for the entire complex (Table 1 and Methods). The Ad11 knob is a trimeric complex composed of three protomers. The asymmetric unit of the crystals contains two Ad11 knob protomers that are located in different trimers, and are each complexed with a single CD46-4D molecule. For each protomer, crystallographic three-fold rotation axes in the P6_3_ space group then generate a trimeric knob structure ligated with three CD46-4D molecules (Fig. 1).

**Table 1:** X-ray data collection and refinement statistics.

**Crystal form**	
Space group	P6_3_
Unit cell dimensions	
a, b, c (Å)	108.16, 108.16, 222.99
α, β, γ (°)	90, 90, 120
**Data collection**	
Wavelength (Å)	1.0
Resolution (Å)^a^	38.82−2.84 (2.99–2.84)
Unique reflections	33537
Redundancy^a^	3.9 (3.4)
Completeness (%)^a^	97.1 (93.8)
R_cryst_ (%)^a^	12.8 (53.1)
I/σI^a^	8.4 (2.0)
**Model statistics**	
Non-hydrogen atoms	7682
** **Protein	7029
Carbohydrates	84
Solvent	378
B-factor overall^b^ (Å^2^)	51.8
Protein	52.0
Carbohydrates	95.3
Solvent	37.1
Resolution (Å)	38.82−2.84
R_work_/R_free_ (%)	21.0/22.9
Number of test reflections	1682
RMS deviations	
Bonds (Å)	0.007
Angles (°)	0.91

a Values for highest resolution shells are given in parentheses.

b composite B-factors containing TLS and residual B factor contributions, mean B_residual_: 32.0 Å^2^.

### Overall organization of the complex

**Figure 2:**
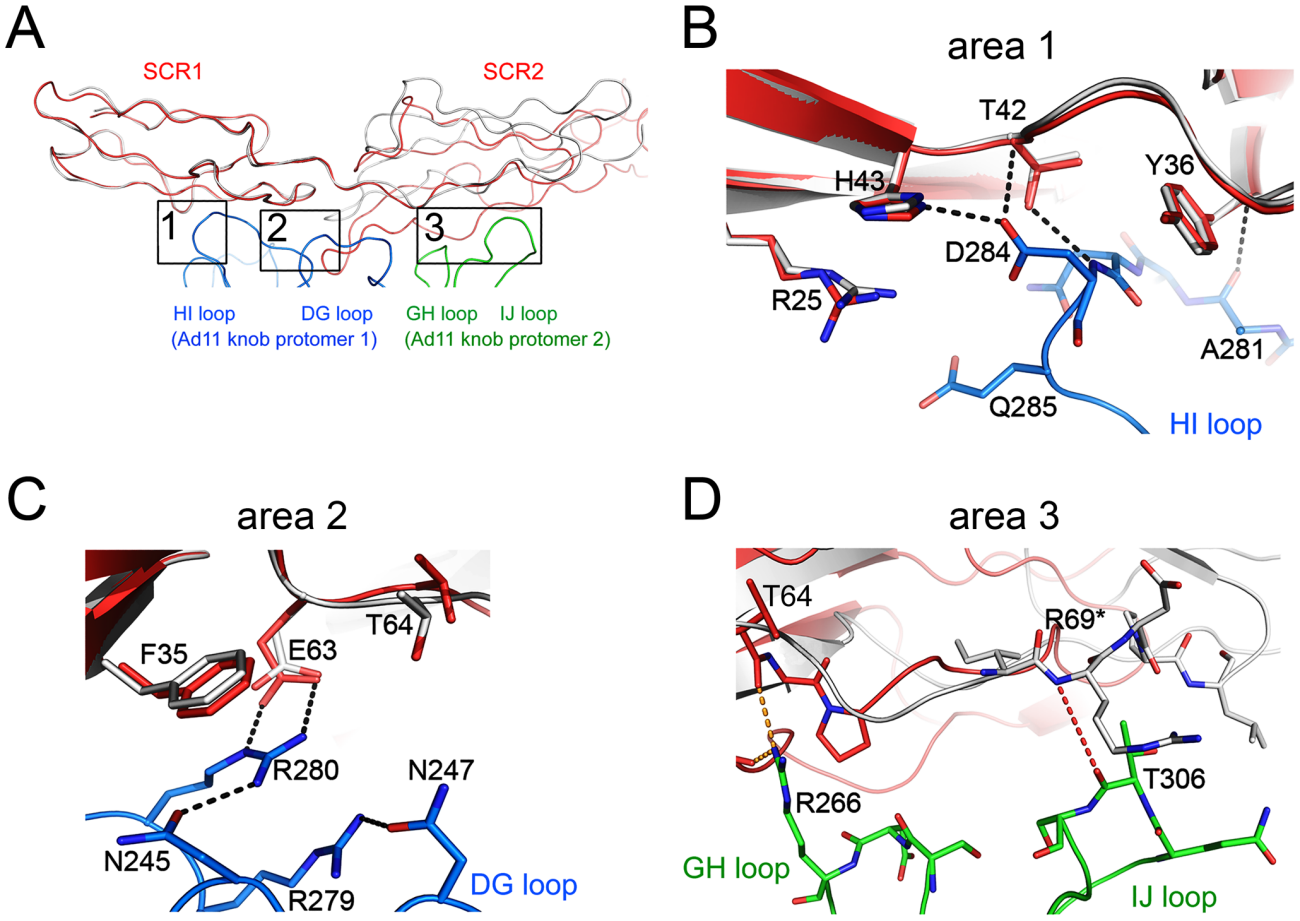
Interactions between Ad11 knob and CD46-4D, and comparison with the structure of the knob in complex with CD46-2D. (A) Overall contact region for one CD46 molecule (red) bound to two Ad11 knob protomers (blue and green). CD46 domains SCR1 and SCR2 contact the extensive loops of the knob protomers. The HI and DG loops are from the blue protomer, whereas the GH and IJ loops are from the green protomer. Superimposed onto the CD46-4D structure (red) is a ribbon drawing of the CD46-2D structure (grey), which was also determined in complex with Ad11 knob [32]. The superposition was performed using Ad11 knob residues only. The three main contact regions (areas 1, 2 and 3) are boxed, and are shown in atomic detail in panels (B), (C) and (D), respectively. Hydrogen bonds and salt bridges (distance<3.5 Å) present in complexes with CD46-2D and CD46-4D are represented with black dashed lines, whereas similar interactions only present in the complex with CD46-2D are shown in orange dashed lines.

At the center of the complex lies the trimeric Ad11 knob structure, which, in support of previous findings [32], [40], engages domains SCR1 and SCR2 but does not interact with domains SCR3 and SCR4 of CD46 (Fig. 1). The SCR1-SCR2 segment adopts a rod-like conformation that is similar but not identical to the one seen in the earlier crystal structure of Ad11 knob in complex with CD46-2D [32] (Fig. 2A). The SCR1 domain and the SCR1-SCR2 interface make nearly identical contacts with the Ad11 knob in both structures, including the central salt bridge between CD46 residue Glu63 and Ad11 knob residue Arg280 (Figs. 2B,C). However, the position and orientation of SCR2 is quite different in the two complexes (Figs. 2A, D). In the Ad11 knob - CD46-2D complex, the SCR2 domain rests on the IJ loop of a second Ad11 knob protomer, forming several contacts including two hydrogen bonds, with the knob. By contrast, SCR2 has moved away from the knob in the Ad11 knob - CD46-4D complex, and the number of contacts have been reduced significantly. As the SCR2 domains are involved in different crystal contacts in the CD46-2D and CD46-4D complexes, we conclude that the interactions of this domain with the Ad11 knob are at least partially determined by crystal packing effects and not crucial for binding. Our data therefore suggest that SCR2 merely rests above the Ad11 knob but does not engage in critical interactions, in line with mutational studies that show that contacts between Ad11 knob and the base of SCR1 are most critical for contact formation [40], [41]. We also conclude that CD46 retains some flexibility at its SCR1-SCR2 interface even when bound to the Ad11 knob.

### Structure and glycosylation of CD46-4D

The CD46-4D chain folds into an elongated structure that is about 115 Å long (Fig. 1). Domains SCR1, SCR2 and SCR3 are arranged in nearly linear fashion, with interdomain angles of 148 and 149 degrees, respectively. However, with an interdomain angle of only 120 degrees between domains SCR3 and SCR4, the SCR4 domain deviates profoundly from the long axis of the protein (Fig. 1). The overall structure of CD46-4D can therefore best be described as resembling a hockey stick, with the N-terminal three domains forming the “shaft” and SCR4 forming the “blade” (Fig. 1). The observed conformation of CD46-4D is nearly identical in both copies of the protein, despite differing crystal contacts.

**Figure 3:**
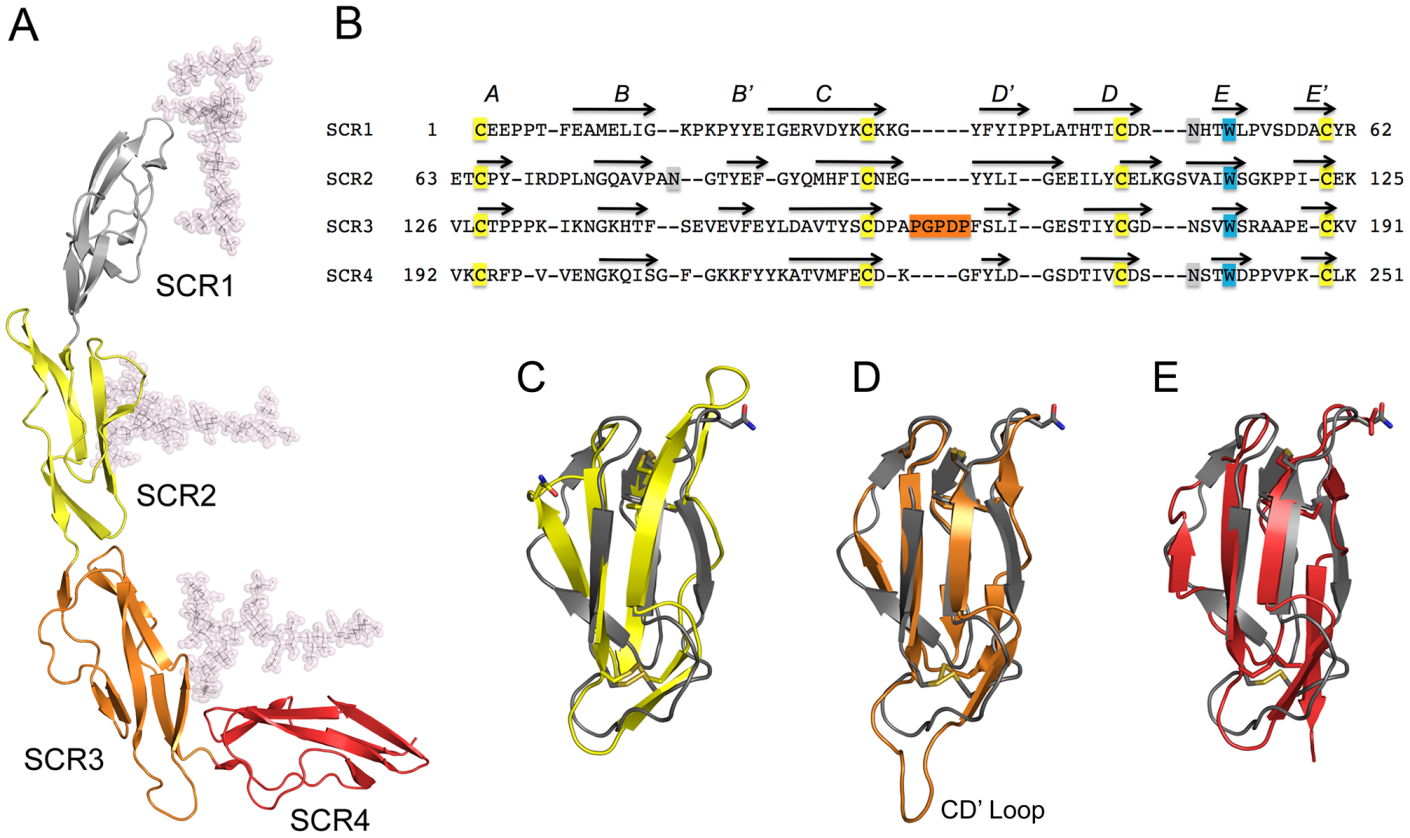
Structure of CD46-4D. (A), Overall structure of CD46-4D, with domains SCR1-SCR4 shown in different colors. The protein carries glycosylation at positions Asn49 (SCR1), Asn80 (SCR2) and Asn239 (SCR4). Although only single NAG residues are visible at Asn49 and Asn80, more extensive glycosylation has been modeled to present a view of the protein that resembles its physiologic state (see Methods). (B) Structural alignment of all four repeats of CD46. The conserved cysteine and tryptophan residues, which are hallmarks of SCR domains, are highlighted in yellow and blue, respectively. The five-residue insertion of the unique CD' loop of SCR3 is shown in orange. Sites of N-linked glycosylation are highlighted in orange. Beta strands are indicated with arrows, and are labeled with letters. The alignment was performed with Modeller (http://salilab.org/modeller/modeller.html) using a gap penalty of 3. (C–E). Superpositions of domains SCR2 (yellow, panel C), SCR3 (orange, panel D) and SCR4 (red, panel E) onto SCR1 (grey). Side chains of conserved cysteine and tryptophan residues of each domain are shown in atomic detail to visualize the agreement of the core domains. Also shown as stick models are the three asparagine residues that carry glycosylation. The unique CD' loop in SCR3 is labeled.

Sequence analysis predicts that CD46-4D carries three N-linked glycans (at Asn49 in SCR1, Asn80 in SCR2, and Asn239 in SCR4). Structures of CD46-2D had shown that Asn49 and Asn80 are glycosylated [31], [32], [33], [34]. In accordance with this, we observe clear electron density for single N-acetyl glucosamine (NAG) residues at both positions, allowing us to incorporate these moieties into the model. Although the electron density at Asn239 is not clear enough to accurately model a carbohydrate into it, its shape and location strongly suggests the presence of a NAG. Thus, all three potential N-linked glycosylation sites of CD46-4D are utilized. Modeling a physiologic glycan structure onto the protein shows that all three glycans would face into the same direction, and would likely shield the concave “inner” side of CD46 entirely from interactions (Fig. 3A and Methods). The STP region of CD46 comprises about 30 amino acids that are not included in our structure. These residues feature sites of O-linked glycosylation and likely serve as a spacer between the base of SCR4 and the membrane. To date, no structural information about this region is available.

### Domain structures and interdomain interfaces in CD46-4D

The prototypical SCR module is primarily composed of four longer β-strands (B, C, D and E) that form a barrel-like structure. The barrel is augmented with a set of smaller β-strands (A, B', D' and E') (Fig. 3B), although not all strands are always present in an SCR. Structural features of SCR1 and SCR2 of CD46, including the domain interface, have been described previously [31], [32]. As expected, the overall folds of the SCR3 and SCR4 modules are quite similar to those of other SCRs such as SCR1 (Figs. 3C–E). The two domains can be superimposed onto SCR1 with low r.m.s. deviations (ranging from 2.2 to 2.8 Å), resulting in nearly identical locations of key features such as the conserved tryptophan side chains and the disulfide bonds that are hallmarks of each SCR (Figs. 3B–E). We note that SCR3 carries a long, almost entirely hydrophobic insertion in its CD' loop (connecting β-strands C and D'), causing this loop to protrude markedly from the domain (Fig. 3D).

**Figure 4:**
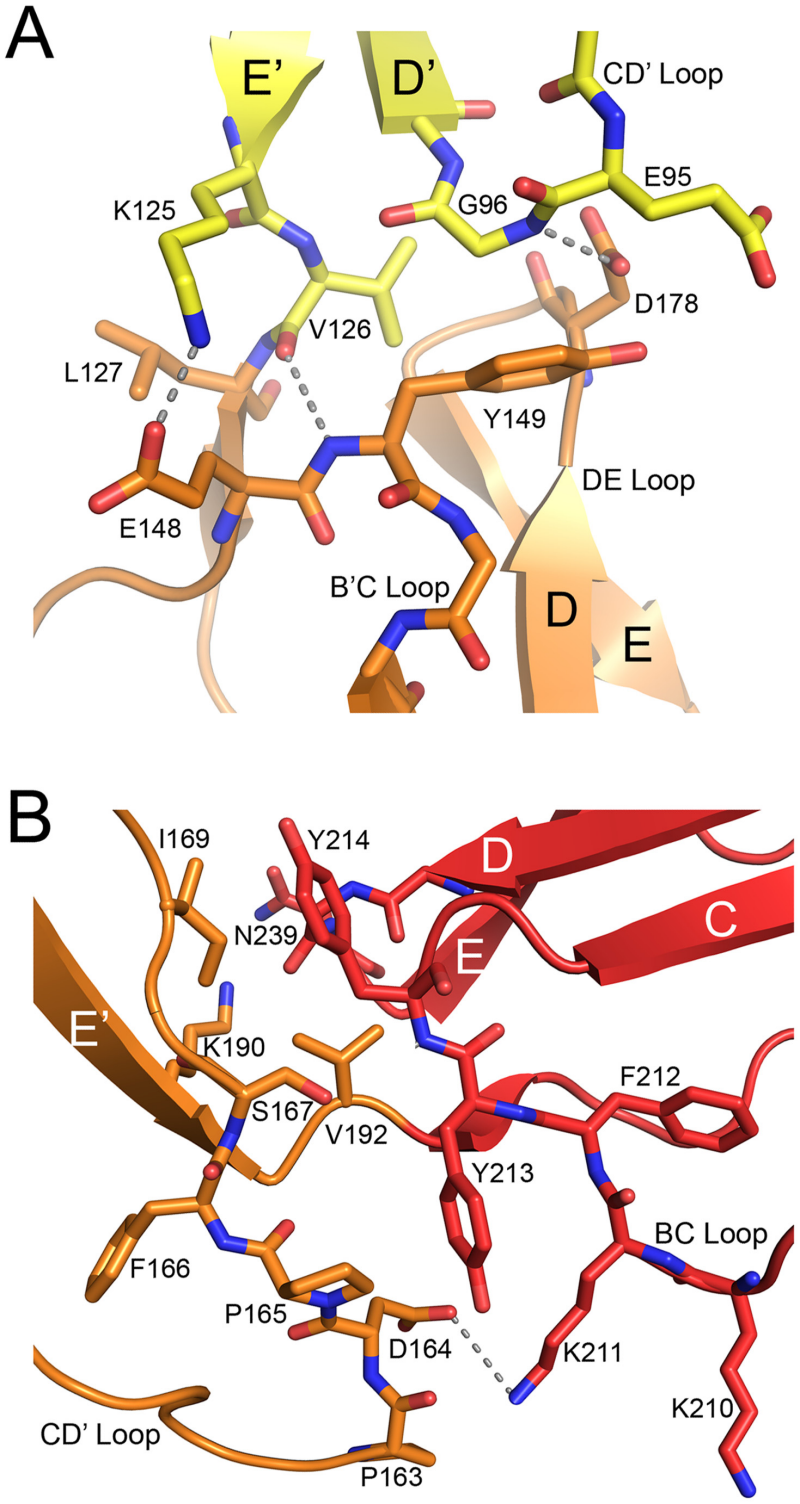
Interdomain interfaces. (A) Interface between domains SCR2 (yellow) and SCR3 (orange). (B) Interface between domains SCR3 (orange) and SCR4 (red). In both cases, residues that participate in contact formation are shown in atomic detail. Hydrogen bonds (distance<3.5 Å) are represented with dashed lines. The orientations of both panels are similar to that shown in Figure 3A.

The interdomain interfaces determine the overall conformation of the protein. Domains SCR2 and SCR3 are stacked together head-to-tail, producing a nearly linear two-domain fragment with interdomain contacts mostly involving the CD' loop of SCR2 and the B'C and DE loops of SCR3 (Fig. 4A). Lys125 makes contacts primarily with SCR2 residues, and Val126 is involved in interactions with SCR3. The interface is stabilized by a hydrogen bond between SCR3 residue Asp178 and the main chain nitrogen of Gly96 in SCR2, and by non-polar contacts between Gly96 and the Tyr149 side chain in SCR3. The interface buries an area of about 480 Å^2^ from solvent, which is comparable to the area buried between domains SCR1 and SCR2 in unliganded CD46 (340 Å^2^) [31].

The interface between SCR3 and SCR4 (Fig. 4B) is unique among the three CD46 interdomain interfaces as it has by far the largest buried surface area (729 Å^2^) and features a profound kink. These characteristics can be directly attributed to the protruding, hydrophobic CD' loop at the base of the SCR3 domain (Fig. 3B,D). Since this loop contains four proline residues, we term it the “proline-rich loop”. The interface is generated by two tyrosines, Tyr213 and Tyr214 at the top of SCR4, that form a cradle-like platform on which the proline-rich loop of SCR3 rests. There are numerous contacts between residues in the proline-rich loop and hydrophobic portions of the two tyrosine side chains as well as SCR4 residue Lys193. The only polar residue in the proline-rich loop, Asp164, lies close to two lysine residues in SCR4, Lys193 and Lys211, and forms weak charge-charge interactions with both. The conformation of the proline-rich loop is incompatible with a more linear arrangement of the SCR3 and SCR4 modules, and since it mediates a large number of interdomain contacts we conclude that this loop is responsible for the profound kink between these two domains. Its unusual length, proline-rich sequence, and key role in interdomain contacts suggest an important function, perhaps by serving as a contact point for complement proteins [37] or by helping to orient the CD46-4D protein at the cell surface (see Discussion).

### Comparison with the structure of FH bound to C3b

**Figure 5:**
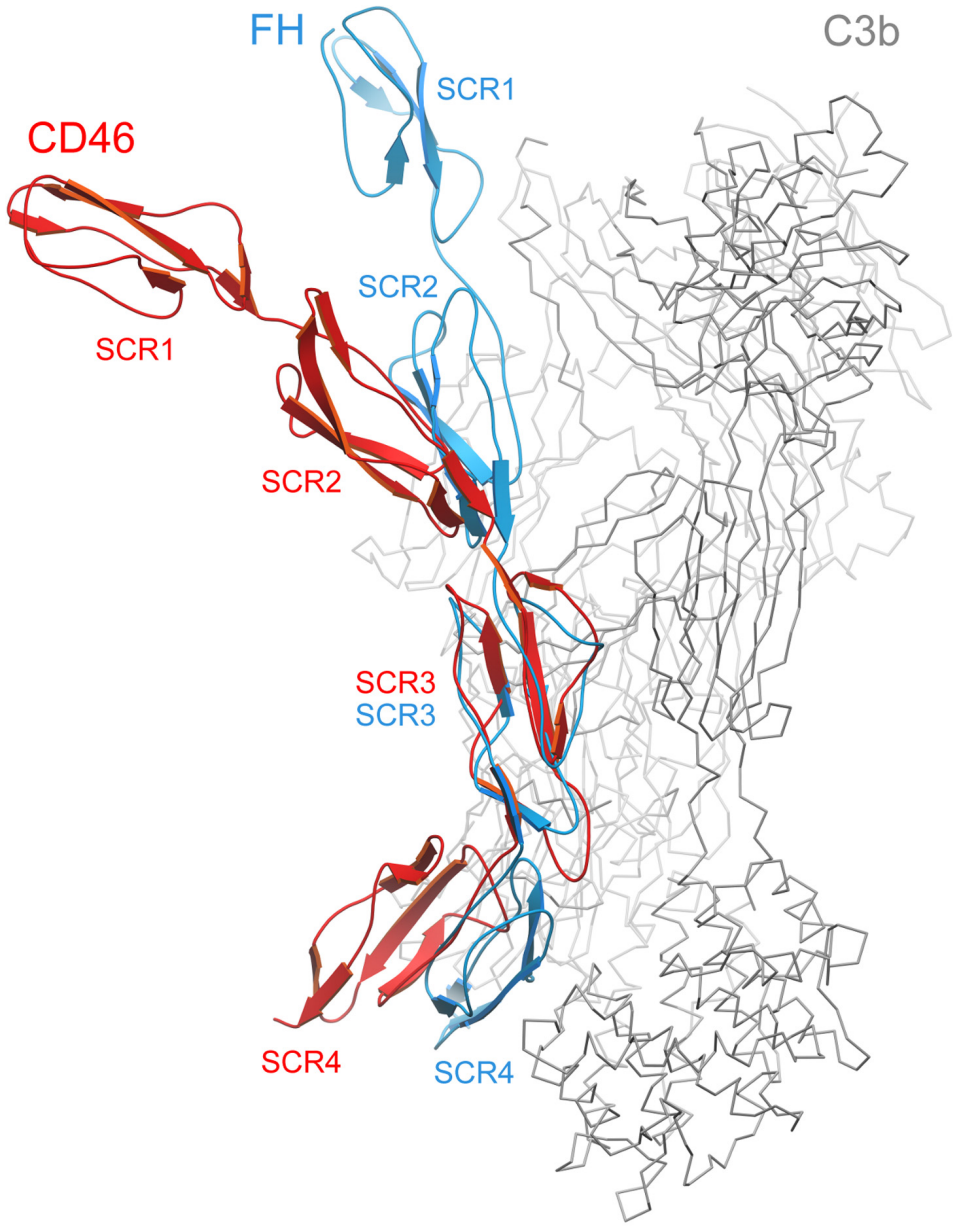
Comparison of CD46-4D with the structure of the N-terminal four repeats of FH. The structures of CD46-4D (red) and FH (PDB code 2WII, blue) [42] were superimposed based on residues in SCR3 only. This yielded an r.m.s. deviation of 1.43 Å for 60 pairs of residues. Shown in grey is the C3b ligand that was crystallized in complex with FH. The individual SCR domains of CD46-4D and FH are labeled in red and blue, respectively.

The crystal structure of C3b in complex with the N-terminal 4 repeats of FH has been reported recently [42]. As C3b serves as a ligand for both CD46 and FH, a comparison of the CD46-4D and FH structures offers useful insights into the location of contact surfaces and overall conformations of proteins constructed from SCR domains. In the C3b-FH complex, domains SCR2, SCR3 and SCR4 of FH engage a large surface that spans the entire side of C3b [42] (Fig. 5). Interestingly, the FH structure also revealed a kink between domains SCR3 and SCR4 at a region that mediates contacts with C3b. With an r.m.s. deviation of 1.43 Å (60 residue pairs), the SCR3 domains of FH and CD46-4D superimpose well. However, this superposition clearly shows that the overall conformations of the four domain segments of FH and CD46 are rather different. The CD46-4D structure is significantly more bent both at the SCR2-SCR3 and SCR3-SCR4 interfaces. It is not known whether the SCR3-SCR4 region is also bent in unliganded FH, or whether the observed bend is caused by contacts with C3b. However, the bend at the SCR3-SCR4 interface of CD46 clearly exists in the absence of ligand and is stabilized by an elongated CD' loop that is unique to the SCR3 domain of CD46 (Fig. 5). As discussed below, the preformed bent CD46 conformation could facilitate binding to C3b.

### Implications for interactions of CD46 with C3b and C4b

**Figure 6:**
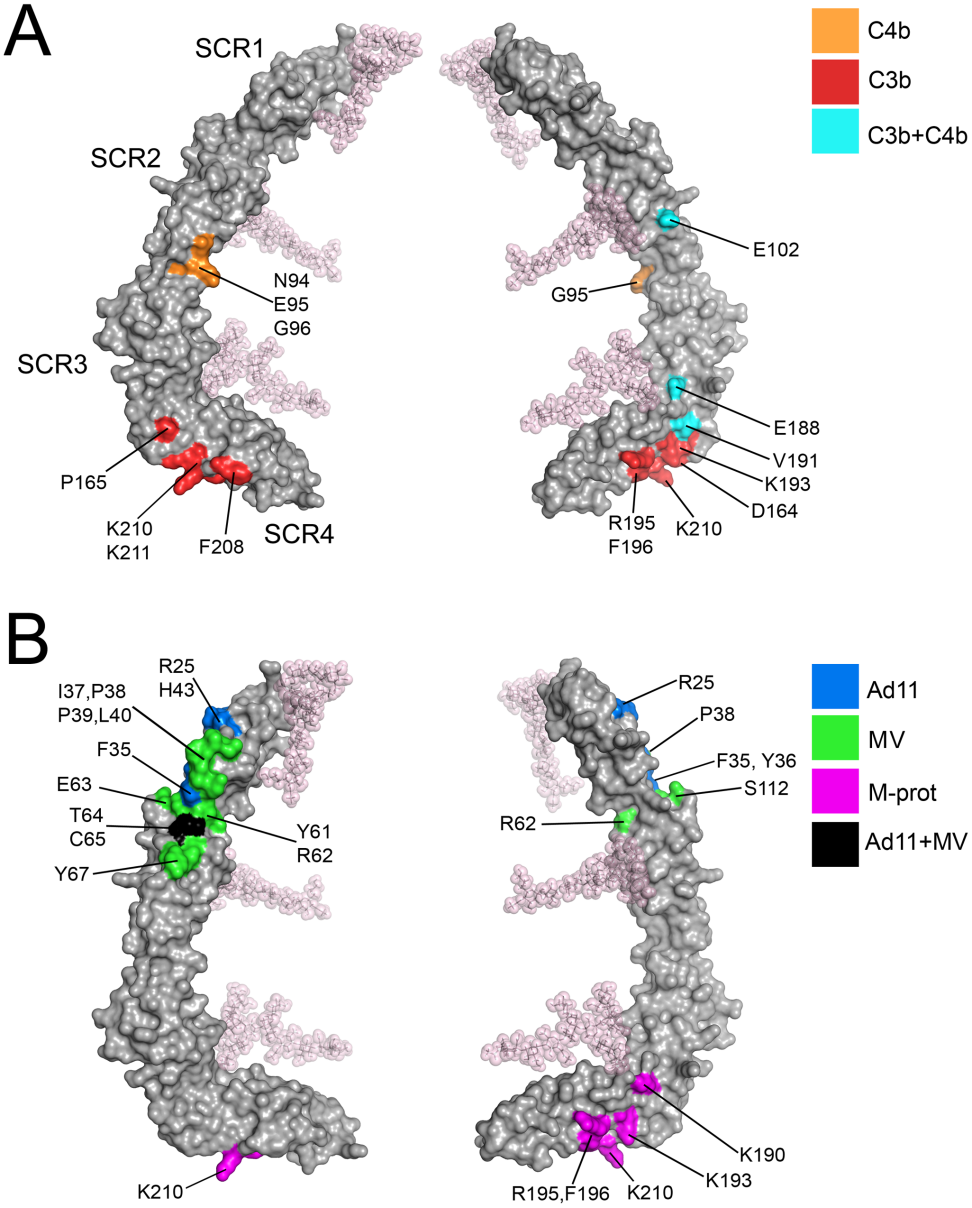
Ligand binding surfaces in the CD46-4D protein. Two views of the CD46-4D structure (grey), differing by 180 degrees along a vertical axis, are shown in each case. (A) Surface representations of CD46-4D, with regions implicated in C3b- (red), C4b- (orange) and C3b + C4b-binding (blue) shown in color [35], [36], [37]. Individual residues are indicated. (B) Surface representations of CD46-4D, with regions known to interact with Ad11 and MV [34] shown in blue and green, respectively. Regions that interact with both viruses are highlighted in black. Residues predicted to contact the *Streptococcus* M protein (M-prot) [38] are shown in purple.

**Figure 7:**
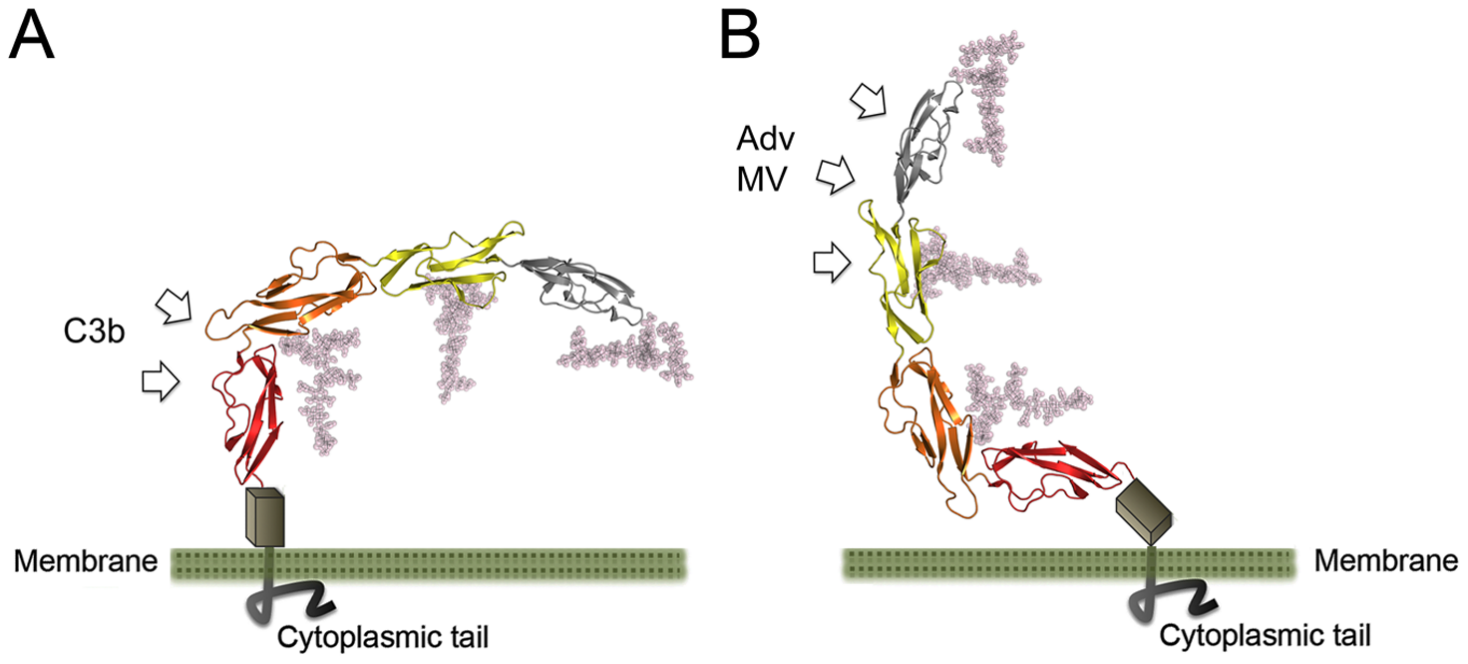
Conformation of CD46 at the cell surface. Two views of the entire CD46 protein, indicating possible orientations on the cell surface. The ribbon drawings show the CD46-4D structure, with domains SCR1-SCR4 colored as in Figure 3. Native glycosylation was modeled as described in the Methods section. The STP region (grey box) comprises about 30 amino acids that are not included in our structure. These residues carry O-linked glycosylation and likely serve as a spacer between the base of SCR4 and the membrane. Arrows indicate likely sites of interaction with C3b (panel A) and viruses (panel B).

Information on C3b and C4b binding to CD46 is primarily based on epitope mapping and mutagenesis experiments, as well as the analysis of molecules lacking specific SCR domains [35], [36], [37]. Taken together, these data indicate (i) that SCR1 is not required for binding C3b or C4b, (ii) that both complement proteins interact with a large portion of the remaining CD46 structure, and (iii) that the binding sites for C3b and C4b are overlapping but distinct. We have mapped all sites that were previously identified as important for binding to C3b and C4b (see Figure 7 in reference [37]) onto the protein surface, excluding amino acids that play a role in function but not direct binding. Intriguingly, the sites for the natural ligands C3b and C4b mostly involve the glycan-free aspects of CD46 and cluster in several smaller areas on SCR2 and SCR3 as well as a large region of SCR4, near the SCR3-SCR4 interface (Fig. 6A). Thus, as was seen in the interactions of CD46 with Adv and MV [32], [33], [34], complement binding appears to be limited to glycan-free regions of CD46.

The CD46 sequence contains three unique regions that are rich in proline residues and that were predicted earlier to interact with C3b/C4b: residues 127-LCTPPPKI-135 at the SCR2-SCR3 interface, residues 159-PAPGPDP-165 in SCR3, and 243-DPPVPKCL-250 in SCR4 [37]. All three regions are partially surface-exposed and available for interactions. The second sequence is especially intriguing as part of it corresponds to the unique insertion in the CD' loop of SCR3 (Fig. 3B,D). This loop is an integral part of the bent SCR3-SCR4 interface (Fig. 4B), and it may therefore play a central role both in determining the overall conformation of CD46 and in mediating interactions with C3b and C4b.

Few amino acid mutations affected binding of C4b to CD46, and not cofactor activity [37]. Amino acids Asn94, Leu95 and Gly96 were found to be relevant only for interactions with C4b, and not C3b. These residues are located within the CD' loop at the base of SCR2, near the SCR2-SCR3 interface, and Gly96 does in fact participate in contacts with SCR3 (Fig. 4A). Thus C4b appears to engage a region closer to the N-terminus of CD46, while also making contact with SCR4 residues.

The extensive C3b-binding epitope covering a large area on SCR4 (Fig. 6A) partially overlaps with a positively-charged region involving a large number of lysine and arginine residues that all lie on one side of SCR4 or near the SCR3-SCR4 interface (Lys190, Lys193, Arg195, Lys203, Lys210, Lys211, Lys224, and Lys251). It is conceivable that some of the basic residues towards the base of SCR4 that are not implicated in C3b binding (e.g., Lys224, Lys251) mediate interactions with negatively-charged membrane lipids.

### Interaction of CD46 with viral and bacterial ligands

Binding sites of Adv and MVH on CD46 have been well characterized by cocrystallization of complexes [32], [33], [34]. Both viruses bind to a similar region of CD46, but they do so by making distinct contacts, with different amino acids. In each case, contacts are limited to SCR1 and SCR2, and they are thus spatially separated from the C3b and C4b binding sites, which do not involve SCR1 at all and are located near the base of the CD46-4D protein (Fig. 6B). Given the large size of the complement proteins, it is nevertheless likely that interaction with either viral protein will directly compete with complement binding.

CD46 also serves as a receptor for *Streptococcus* on keratinocytes [14]. Interactions are mediated by the streptococcal surface protein, M, a long, filamentous protein that is also able to engage other members of the RCA family. Using domain exchange experiments and chimeric CD46/CD55 molecules, Giannakis et al. [38] showed that binding of the M protein is dependent only on domains SCR3 and SCR4 of CD46. Sequence comparison of CD46 with other RCA family members for which M protein binding has been mapped to individual residues [43], [44] suggests that M protein primarily interacts with a region of SCR4 that partially overlaps with binding sites for C3b and C4b (compare Fig. 6B with Fig. 6A). However, C3b-mediated complement activity was detectable even after addition of M protein [38], indicating that the binding sites for C3b and M protein are not identical.

The binding sites of *Neisseria* and HHV-6 have been mapped to individual domains only. The SCR3 and STP domains of CD46 are required to mediate adherence of Neisseria [17], while interactions of HHV-6 with CD46 depend on repeats SCR2 and SCR3 [19]. In both cases, therefore, interactions appear to be distant from the binding sites for Adv and MV, and they are also expected to compete with the binding of C3b or C4b to CD46.

## Discussion

Precise regulation of immune defense mechanisms is essential to protect host tissue from injury. This is achieved in part by mechanisms that prevent the inappropriate activation of complement on autologous tissues. The RCA family of proteins plays a key role in this process by interacting with fragments of complement proteins C3 and C4. The CD46 protein inhibits complement activation by binding separately to C3b and C4b and promoting their proteolytic inactivation by factor I [4]. In addition, CD46 also serves as the cell attachment receptor for a number of human pathogens [22].

We have determined the three-dimensional structure of all four SCR domains of CD46, which constitutes the bulk of the extracellular region of this cell surface receptor protein, in complex with the Ad11 knob. The conformation of CD46-4D resembles a hockey stick, with an unexpected bend between domains SCR3 and SCR4. This bend can be attributed to a unique five-residue insertion into the CD' loop of SCR3 (Figs. 3B,D). The insertion is not compatible with a linear arrangement of the SCR3-SCR4 interface but instead provides a platform that stabilizes the bent structure. The smaller SCR1-SCR2 interface possesses some flexibility [31], and flexibility may also be a feature of the similarly-sized SCR2-SCR3 interface. However, our structure suggests that the SCR3-SCR4 interface has little, if any, flexibility as it has a much larger buried surface area, exhibits low temperature factors, and contains many rigid amino acids. The role of the CD' loop in SCR3 thus appears to be in forming a brace that molds the SCR3-SCR4 unit of CD46 into a bent conformation. Inspection of sequences of RCA family members shows that no homolog of CD46 contains a similarly elongated and hydrophobic loop [45].

Previous studies identified a number of residues that play a role in mediating interactions of CD46 with its many ligands. Our structure now places these data in proper context by displaying CD46 ligand binding surfaces on the extracellular portion of the molecule. Interactions of CD46 with Adv and MV are exclusively mediated by the SCR1-SCR2 region, and these interactions have been described earlier [32], [33], [34]. Our analysis indicates that interactions with C3b and C4b involve several regions on domains SCR2, SCR3 and SCR4. These regions are located on the convex surface of the curved receptor molecule, and they are devoid of glycosylation. The most extensive binding site for C3b is located on one side of SCR4, and appears to depend on a number of charged residues. This region partially overlaps with a binding site for C4b. Smaller contact regions for both C3b and C4b are located at the base of SCR2, near the SCR2/SCR3 interface. Thus, the large C3b and C4b proteins probably contact a significant portion of the surface of CD46 that is defined by the SCR2-SCR4 fragment, similar to the contacts observed in the recent crystal structure of the N-terminal four SCR domains of FH in complex with C3b [42] (Fig. 5). Therefore, the bent conformation of CD46 could be a highly significant determinant for the recognition of complement proteins. In contrast to soluble FH, which contains 20 SCR domains, the CD46 protein is much smaller and attached to the membrane. The presence of a preformed bend in the protein conformation could facilitate association of complement proteins to CD46 on the cell surface, reducing, or perhaps eliminating, the requirement for domain rearrangements during C3b and C4b binding.

The structure reported here does not include the short STP region, which connects SCR4 to the single transmembrane spanning sequence of CD46. We can therefore not provide a definitive view of how the CD46 molecule is arranged on the cell surface. The proline-rich nature of the STP region suggests that it has limited flexibility, perhaps serving as a stalk that provides some distance between SCR4 and the membrane surface. Two extreme possibilities for the conformation of CD46 on the cell surface can be envisaged (Fig. 7). In one of these, the SCR4 domain and the STP region project vertically from the cell surface, generating a protein arrangement in which the glycans face toward the membrane and the N-terminal SCR1 domain is near the cell surface (Fig. 7A). Interactions of the glycans with the membrane could help to orient the molecule on the cell surface, with the glycan-free region being highly accessible for interactions with even large ligands such as complement proteins C3b and C4b. Moreover, the proximity of the SCR1 domain to the membrane, which serves as the main contact point for Adv and MV, would facilitate penetration of the cell membrane by those viruses, and in particular fusion of MV and cell membranes. In the second scenario, the STP region is bent, and the SCR4 lies more or less parallel to the cell surface (Fig. 7B). The SCR1-SCR2 region would project into solution, and would readily be available for interactions with Adv and MV, but also more distant from the cell surface. If such an arrangement were to exist at the cell surface, it might preclude binding of C3b (and perhaps C4b) to CD46 as the predicted sites for C3b binding on SCR4 would face towards the membrane, and thus would not be easily accessible to the large C3b protein.

In order to expose the complement binding sites on SCR4, CD46 would need to adopt a conformation in which the SCR1 domain would be close to the membrane (Fig. 7A). Multivalent interaction of the Adv knob with CD46 in this conformation would require either movements within the STP region toward an alternative CD46 conformation (Fig. 7B), which could be limited by the proline rich nature of this region, or some plasticity in the cell membrane for virus binding to multiple receptor molecules. Trimeric binding of the knob to CD46 molecules adopting a conformation similar to that shown in Fig. 7B could be accomplished in concave membrane microdomains. Alternative splicing variants of the STP region could influence the orientation of the CD46 molecule on the cell surface. It has been shown that alternative splicing in this region has significant implications for complement regulatory function [46], [47] as well as MV binding and fusion [47], [48]. The overall structure of the CD46 extracellular region presented here differs drastically from earlier models that pictured CD46 as an elongated, rod-like structure, and suggests a more dynamic conformation of this receptor molecule on the cell surface.

## Materials and Methods

### Protein expression and purification

A cDNA encoding residues 1 to 286 of the CD46 precursor protein was subcloned into the expression vector pBJ5-GS [49]. This vector was transfected into CHO Lec 3.2.8.1 cells [50], and stable cell clone transfectants secreting the CD46-4D protein to the culture medium were selected with methionine sulfoximine, a glutamine synthetase inhibitor. Transfected cells were cultured in Ex-Cell 302 medium (JRH Biosciences) supplemented with 100 µM methionine sulfoximine, GS supplement (JRH Biosciences), 50 units/ml penicillin G, 50 mg/ml streptomycin, 7.5 mM HEPES at pH 7.3 and 1% dialyzed fetal bovine serum. After harvesting, the culture supernatant was centrifuged and filtered. CD46-4D was then purified by Concanavalin A affinity chromatography (Con A Sepharose, GE Healthcare), gel filtration (Superdex 200, GE Healthcare), and anion exchange chromatography (MonoQ, GE Healthcare).

Ad11 fiber knob amino acids 118–325 were expressed in *E. coli* Rosetta2 (DE3) cells and purified via nickel affinity chromatography and gel filtration, as described earlier [32]. The complex was formed by incubating both proteins at 4°C for 2 hrs. A 1.2 molar excess of CD46-4D was used, based on the earlier observation that one trimeric knob can bind three CD46 ligands [32]. Separation of the complex from excess, unbound CD46-4D was performed by size exclusion chromatography (Superdex 200 HR column (GE Healthcare, Uppsala, Sweden) in gel filtration buffer containing 20 mM HEPES, 150 mM NaCl at pH 7.4.

### Crystallization and structure determination

Well-diffracting plate-like crystals of Ad11 knob in complex with CD46-4D were obtained at 4°C using a precipitant solution containing 20% polyethylene glycol 1000, 0.2 M ammonium phosphate at pH 8.0 with the use of a microseeding protocol [51]. Poorly diffracting crystals grown at 20°C in 20% PEG 6000, 200 mM ammonium phosphate pH 8.0 were used for seeding. Crystals belong to space group P6_3_, with two copies of Ad11 knob protomers and two CD46-4D chains present in the asymmetric unit. The crystals were flash frozen in liquid nitrogen using precipitant solution supplemented with 25% PEG 200 for cryogenic protection. Diffraction data were collected at the Swiss Light Source (beam line X06SA) and ESRF (beam line BM14). Diffraction images were processed using XDS [52] and SCALA [53], producing a data set that extends to 2.84 Å with good statistics. The structure determination was carried out by molecular replacement with Phaser [54]. Coordinates for the Ad11-knob protomer as well as the SCR1 and SCR2 domains of CD46 [32] were used independently as search models, after removal of surface loops that had elevated temperature factors in each case. This strategy produced two clear solutions for the complex, indicating the presence of two copies of Ad11 knob protomers and two CD46 molecules in the asymmetric unit. After initial rigid body refinement using NCS restraints in Phenix [55], 2Fo-Fc and Fo-Fc difference electron density maps revealed the location of the SCR3-SCR4 portions in both copies of CD46. These domains were then included into the model. The structure was built using Coot [56] and O [57], and refined using REFMAC5 [58], Phenix [55] and Autobuster [59]. The entire model could be built with the exception of residues 81–84 of SCR2 in chain D, which probably have multiple conformations in the crystal. Coordinates and structure factor amplitudes have been deposited with the Protein Data Bank (PBD ID code 3O8E). Figure 5 was prepared using Molscript [60], all other figures were made with PyMol [61]. Superpositions were done with LSQKAB [53] and the SSM routine in Coot [56].

### Glycan modeling

CD46-4D has three N-linked glycosylation sites, at Asn49, Asn80 and Asn239. In order to produce a realistic estimate of size and distribution of the glycan structure of native human CD46-4D, we used the GlyProt online server [62] and modeled hybrid and complex glycans linked to the three Asn residues with NAG electron density.
